# Supplementary material for: Association of Dengue Virus Serotypes 1&2 with Severe Dengue Having Deletions in Their 3′Untranslated Regions (3′UTRs)
Source: Microorganisms. 2023 Mar 6;11(3):666. doi: 10.3390/microorganisms11030666 (PMC10057630; doi:10.3390/microorganisms11030666)
Supplement: Supplementary file 1 [file microorganisms-11-00666-s001.zip › microorganisms-2150466-supplementary.pptx]

## Slide 1
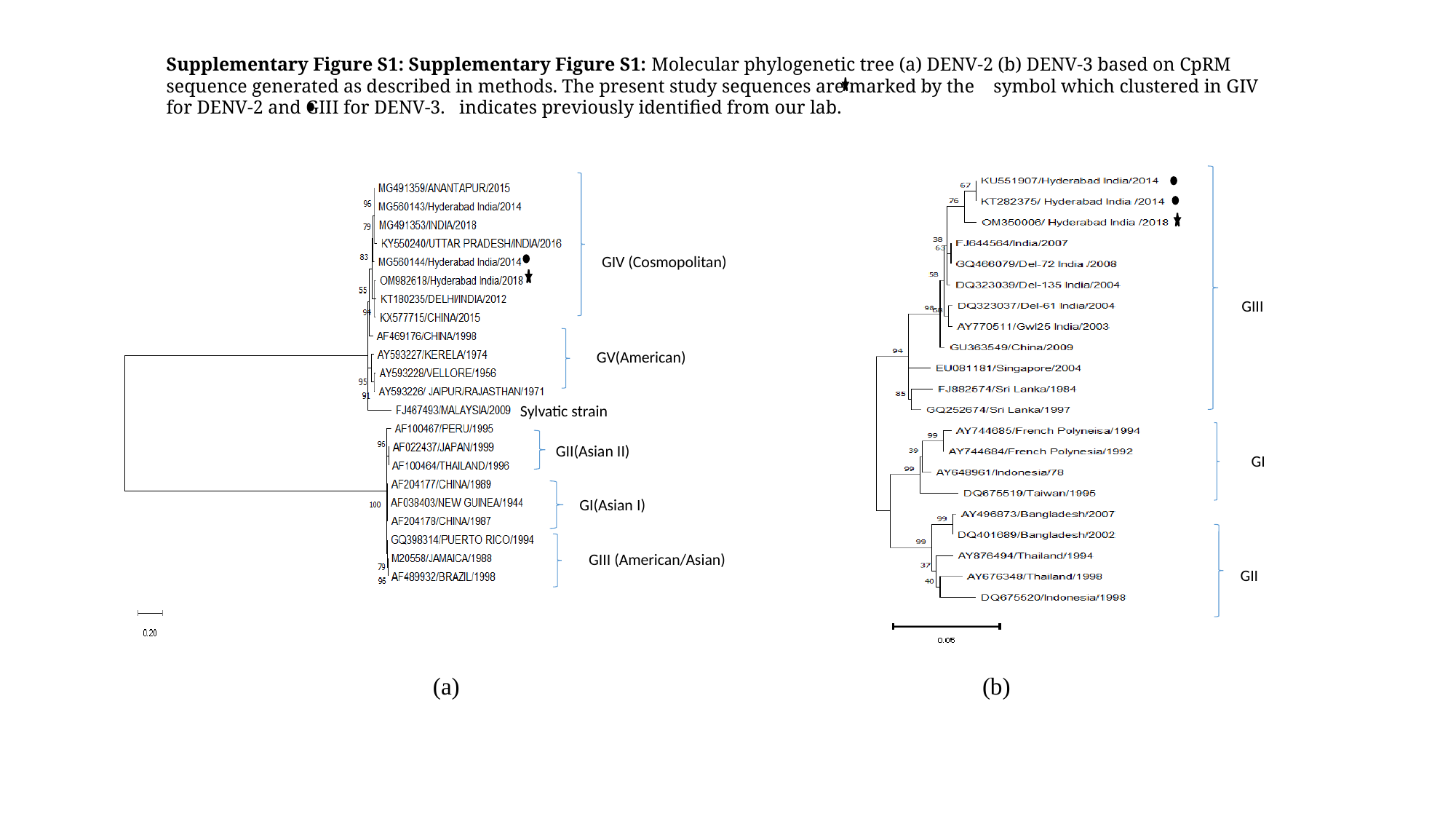

Supplementary Figure S1: Supplementary Figure S1: Molecular phylogenetic tree (a) DENV-2 (b) DENV-3 based on CpRM sequence generated as described in methods. The present study sequences are marked by the symbol which clustered in GIV for DENV-2 and GIII for DENV-3. indicates previously identified from our lab.
GIV (Cosmopolitan)
GIII
GV(American)
Sylvatic strain
GII(Asian II)
GI
GI(Asian I)
GIII (American/Asian)
GII
(a)
(b)

## Slide 2
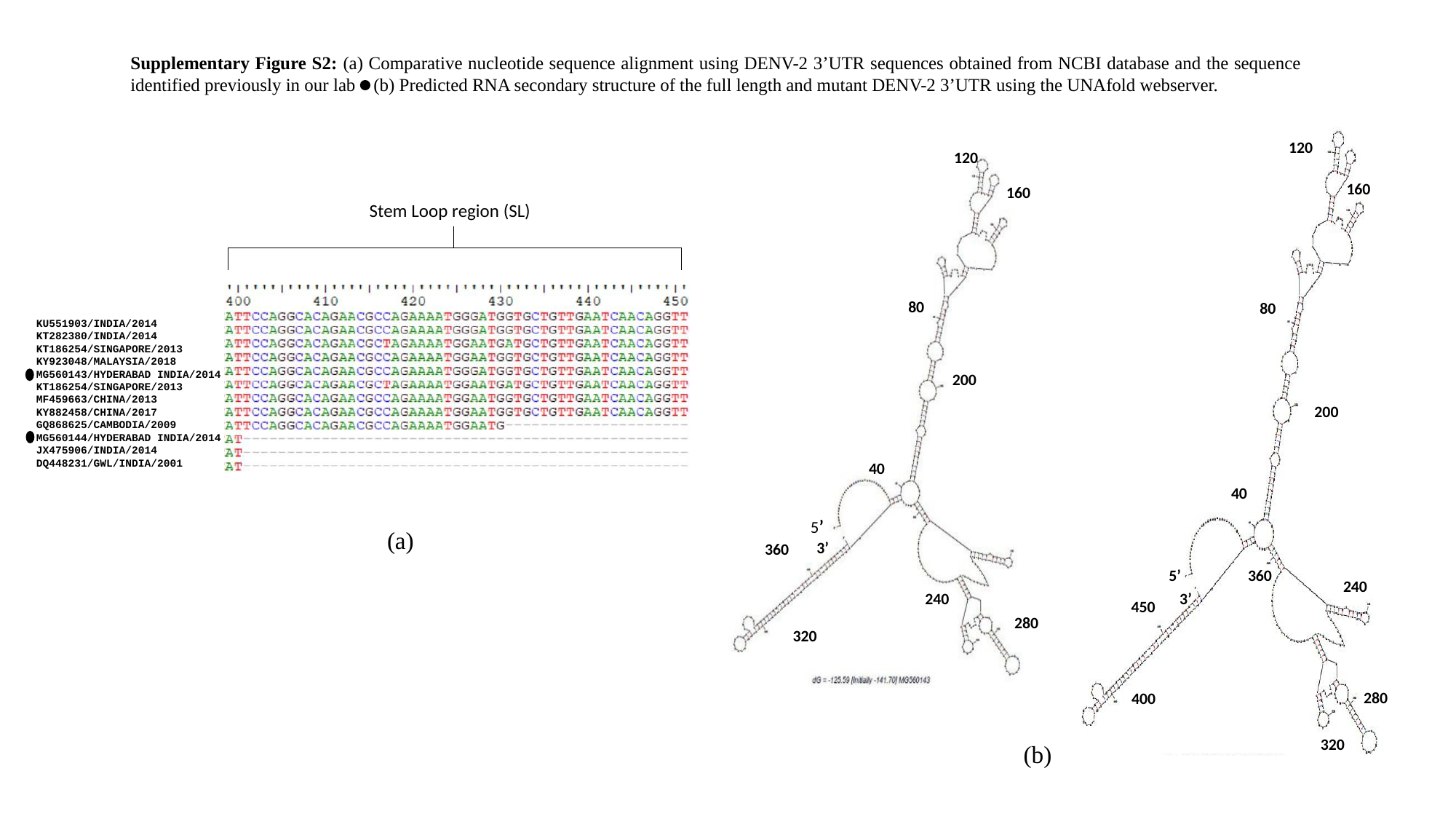

Supplementary Figure S2: (a) Comparative nucleotide sequence alignment using DENV-2 3’UTR sequences obtained from NCBI database and the sequence identified previously in our lab (b) Predicted RNA secondary structure of the full length and mutant DENV-2 3’UTR using the UNAfold webserver.
120
120
160
160
Stem Loop region (SL)
80
80
KU551903/INDIA/2014
KT282380/INDIA/2014
KT186254/SINGAPORE/2013
KY923048/MALAYSIA/2018
MG560143/HYDERABAD INDIA/2014
KT186254/SINGAPORE/2013
MF459663/CHINA/2013
KY882458/CHINA/2017
GQ868625/CAMBODIA/2009
MG560144/HYDERABAD INDIA/2014
JX475906/INDIA/2014
DQ448231/GWL/INDIA/2001
200
200
40
40
5’
(a)
3’
360
5’
360
240
240
3’
450
280
320
280
400
320
(b)
